# Proxy gene-by-environment Mendelian randomization study of the association between cigarette smoking during pregnancy and offspring mental health

**DOI:** 10.1093/ije/dyad022

**Published:** 2023-03-01

**Authors:** Hannah M Sallis, Robyn E Wootton, George Davey Smith, Marcus R Munafò

**Affiliations:** Centre for Academic Mental Health, Population Health Sciences, Bristol Medical School, University of Bristol, Bristol, UK; MRC Integrative Epidemiology Unit (IEU) at the University of Bristol, Bristol, UK; MRC Integrative Epidemiology Unit (IEU) at the University of Bristol, Bristol, UK; Nic Waals Institute, Lovisenberg Diaconal Hospital, Oslo, Norway; MRC Integrative Epidemiology Unit (IEU) at the University of Bristol, Bristol, UK; MRC Integrative Epidemiology Unit (IEU) at the University of Bristol, Bristol, UK; School of Psychological Science, University of Bristol, Bristol, UK; NIHR Biomedical Research Centre, University Hospitals Bristol NHS Foundation Trust and University of Bristol, Bristol, UK

**Keywords:** Mendelian randomization, smoking, depression, schizophrenia

## Abstract

**Background:**

Smoking prevalence is higher among individuals with schizophrenia or depression, and previous work has suggested this relationship is causal. However, this may be due to dynastic effects, for example reflecting maternal smoking during pregnancy rather than a direct effect of smoking. We used a proxy gene-by-environment Mendelian randomization approach to investigate whether there is a causal effect of maternal heaviness of smoking during pregnancy on offspring mental health.

**Methods:**

Analyses were performed in the UK Biobank cohort. Individuals with data on smoking status, maternal smoking during pregnancy, a diagnosis of schizophrenia or depression, and genetic data were included. We used participants’ genotype (rs16969968 in the *CHRNA5* gene) as a proxy for their mothers’ genotype. Analyses were stratified on participants’ own smoking status in order to estimate the effect of maternal smoking heaviness during pregnancy independently of offspring smoking.

**Results:**

The effect of maternal smoking on offspring schizophrenia was in opposing directions when stratifying on offspring smoking status. Among offspring of never smokers, each additional risk allele for maternal smoking heaviness appeared to have a protective effect [odds ratio (OR)* *=* *0.77, 95% confidence interval (CI) 0.62 to 0.95, *P = *0.015], whereas among ever smokers the effect of maternal smoking was in the reverse direction (OR = 1.23, 95% CI 1.05 to 1.45, *P = *0.011, *P*_interaction_* *<0.001). There was no clear evidence of an association between maternal smoking heaviness and offspring depression.

**Conclusions:**

These findings do not provide clear evidence of an effect of maternal smoking during pregnancy on offspring schizophrenia or depression, which implies that any causal effect of smoking on schizophrenia or depression is direct.

Key Messages

- Smoking prevalence is higher among individuals with schizophrenia and depression; however, the role of dynastic effects (for example, reflecting the effects of their maternal smoking during pregnancy, rather than a direct effect of their own smoking) in this relationship is unclear.
- Proxy gene-by-environment Mendelian randomization analyses can be used to investigate whether there is a causal role of maternal smoking during pregnancy on offspring mental health.
- We found no clear evidence for a causal role of maternal smoking during pregnancy, suggesting that any causal role of smoking on schizophrenia and depression is direct.

## Introduction

Smoking prevalence is higher among individuals with schizophrenia and depression compared with the general population.^1–5^ One argument for this association is the ‘self-medication’ hypothesis that suggests individuals may smoke in order to alleviate symptoms of mental illness or side effects of medication.^6^^,^^7^ However, other studies have suggested that the relationship may also run in the opposite direction, with smoking being a risk factor for subsequent mental illness.^8–11^

Previous work by Wootton and colleagues used a Mendelian randomization approach and found evidence of a causal effect of own smoking on both schizophrenia and depression.^8^ However, this study design did not exclude possible dynastic effects: this is where the expression of parental genetics via parental phenotype directly influences offspring phenotype.^12^ In this case, maternal rs16969968 genotype will influence maternal smoking heaviness, which could directly affect offspring mental health. If this pathway acts via a route that does not include offspring smoking, then this could result in a violation of one of the fundamental assumptions of Mendelian randomization.^13^ Under this assumption there should be no confounding of the relationship between the genetic variant and the outcome.

Supporting a possible dynastic effect, maternal smoking during pregnancy has been associated with adverse offspring outcomes, including depression and severe mental illness.^9^^,^^14–17^ Prenatal smoke exposure can be a potential risk to the developing fetus, as nicotine can cross the placenta and fetal-blood brain barrier. It has also been suggested that prenatal smoking can result in dysregulation of the fetal hypothalamic-pituitary-adrenal (HPA) axis, which may be linked to the development of psychopathology.^18^ The causal effects of an individual’s own smoking compared with maternal smoking are difficult, but important, to separate.

However, evidence relating to the effects of maternal smoking during pregnancy on offspring mental health is mixed.^14–16^^,^^19–21^ The majority of this evidence to date uses observational data, from which it is difficult to infer causality with confidence.^9^ A negative control study carried out in the Avon Longitudinal Study of Parents and Children (ALSPAC) did find some evidence of a causal effect of *in utero* tobacco exposure on psychotic experiences in adolescence.^20^ Conversely, a cross-cohort and negative control study of maternal smoking in pregnancy and offspring depression suggested that observed associations could reflect residual confounding relating to parental characteristics.^21^

In this study, we used data from the UK Biobank study and a proxy gene-by-environment (GxE) Mendelian randomization approach to investigate whether there is evidence for a causal effect of maternal heaviness of smoking during pregnancy on offspring schizophrenia or depression.^22^ We used genetic data from participants at the rs16969968 locus in the *CHRNA5* gene as a proxy for maternal genotype, which was unavailable in the study. The use of a genetic variant as a proxy for maternal smoking means that our analysis should be less subject to the problems of confounding and reverse causation that conventional observational studies are often subject to.^13^^,^^23^^,^^24^ This approach also enables us to investigate the effects of maternal smoking during pregnancy on offspring outcomes even when maternal genetic information is not available. This proxy GxE method has previously been validated by replicating the effect of maternal smoking during pregnancy on birthweight.^22^

## Methods and measures

### Sample

The UK Biobank Study is a large population cohort consisting of around 500 000 individuals recruited in the UK between 2006 and 2010. Eligible individuals were aged between 40 and 69 years at time of recruitment and living within 25 miles of an assessment centre (22 assessment centres were based across England, Wales and Scotland). Approximately 9.2 million individuals were invited to take part, with around 5.5% of these participating in the baseline assessment.^25^ Informed consent was obtained from study participants by UK Biobank. UK Biobank received ethics approval from the Research Ethics Committee (REC reference for UK Biobank is 11/NW/0382).

### Smoking status

Maternal smoking during pregnancy was reported by UK Biobank participants (the offspring) in response to the question ‘Did your mother smoke regularly around the time when you were born?’ (variable: 1787). Offspring smoking status was categorized as never or ever (former or current) smokers according to participants’ self-reported smoking status at recruitment (variable: 20116).

### Offspring schizophrenia diagnosis

Diagnoses of schizophrenia were derived using a combination of approaches including the International Classification of Diseases, Tenth Revision (ICD-10) diagnoses (variable 41202 and 41204, category F20) and date of diagnosis (variable: 130874), identified via a combination of sources including death registers, primary care sources, hospital admissions data and self-report. We also included self-reported diagnoses using the non-cancer illness item (variable: 20002, category for schizophrenia: 1289). Responses to this item were derived from the verbal interview conducted at the initial assessment. In total, we identified 528 (0.18%) individuals with a diagnosis of schizophrenia based on the date of diagnosis item (*n *= 526), ICD-10 codes (*n *= 336) and the non-cancer illness item (*n* = 276). All other participants in the analysis sample were categorized as controls (*n *= 288 165).

### Offspring depression diagnosis

We used two definitions of depressive disorder. The first was a stricter definition of depression as measured by responses to the online mental health questionnaire (MHQ; variable: 20126), referred to from now on as major depressive disorder (MDD). The second was less specific about the type of depression and was measured by responses to both the self-reported diagnoses using the non-cancer illness item (variable: 20002, category for depression: 1286) and the ICD-10 diagnoses (variable: 41202 and 41204, categories: F32 or F33), referred to from here on as depression. A total of 18 704 individuals reported a diagnosis of MDD using the MHQ (27.1%) and 20 901 (7.24%) individuals reported a diagnosis of depression based on the non-cancer illness item (*n* = 16 318) and the ICD-10 item (*n* = 8125).

### Genetic instrument for maternal smoking

Maternal genotype is unavailable in UK Biobank, so participants’ genotype was used as a proxy. There are several potential variants in the *CHRNA5-A3-B4* gene cluster that could have been used as a proxy for smoking heaviness (e.g. rs16969968, rs1051730, rs2036527, rs17486278, rs17487223), and these are all highly correlated.^26^ A recent genome-wide association study of tobacco use identified variants within the CHRNA5-A3-B4 gene cluster that are associated with smoking initiation.^27^ However, these variants are not correlated with the rs16969968 single nucleotide polymorphism (SNP), and this gene cluster appears to have distinct influences on smoking initiation and heaviness.

We chose to use the rs16969968 variant in the *CHRNA5-A3-B4* gene cluster as a genetic instrument for smoking heaviness. This variant is a missense mutation in the α-5 subunit and appears to have functional significance.^26^ This variant is located within the nicotinic acetylcholine receptor gene cluster and causes an amino acid change that is associated with a reduced response to nicotine.^26^^,^^28^ The rs16969968 SNP is therefore likely to be the functional variant associated with smoking heaviness. Each additional copy of the A allele is associated with smoking one additional cigarette per day on average.^29^ Analyses were restricted to individuals of White British descent to avoid introducing bias due to population stratification.^30^

### Statistical analysis

**Figure 1:**
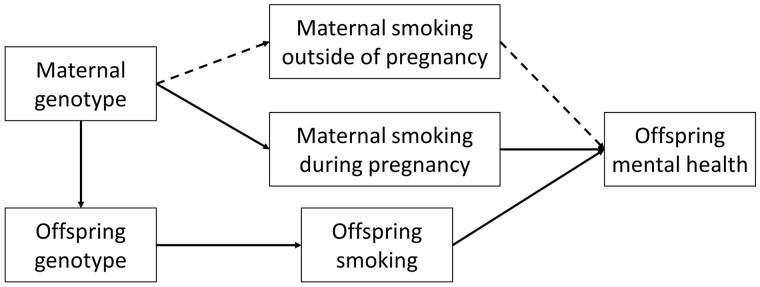
Proxy gene-by-environment Mendelian randomization conceptual framework—offspring genotype is used as a proxy for maternal genotype and analyses are stratified on maternal substance use during pregnancy. Maternal substance use outside of pregnancy could influence offspring mental health via alternative pathways (e.g. oocyte quality or passive smoking in the case of smoking heaviness—shown by dashed lines). Offspring substance use may also influence offspring mental health, and analyses are therefore also stratified on offspring substance use. Adapted from Yang Q, Millard LAC and Davey Smith G^22^

We used a proxy GxE Mendelian randomization approach as described in Yang and colleagues,^22^ to test for causal effects of maternal smoking on offspring mental health, where offspring genotype is used as a proxy for maternal genotype. Logistic regression analyses were performed to investigate the association between rs16969968 and diagnosis of schizophrenia or depression. It is possible that offspring genotype could influence their mental health via both maternal and their own smoking heaviness. Analyses were therefore stratified on participants’ own smoking status and adjusted for the top 10 principal components of ancestry, birth year and gender, in order to estimate the effect of maternal smoking heaviness during pregnancy independently of offspring smoking (Figure 1).

As a sensitivity analysis, we re-ran the analysis excluding individuals who were heterozygous at rs16969968, to ensure we knew which allele was received from the mother. Diagnoses of schizophrenia are much lower in UK Biobank (0.19%) than in the general population (lifetime prevalence estimates ∼1%),^31^ which could mean that these findings are at risk of distortion by collider bias.^32^ Depression is under less selection in UK Biobank, and we used two distinct phenotype definitions, which should minimize the risk of collider bias. In order to investigate the extent of collider bias due to selection of the offspring schizophrenia phenotype, we explored whether the association between diagnosis and known risk factors for schizophrenia was as expected in our sample. We expected an inverse association between both birthweight and education with schizophrenia diagnosis, and a positive association between genetic liability to schizophrenia and schizophrenia diagnosis.^33–35^

## Results

There were 288 693 individuals in UK Biobank with relevant data on smoking status, maternal smoking during pregnancy, self-reported diagnosis of schizophrenia or depression and rs16969968 genotype. When using the MDD diagnosis from the touchscreen questionnaire, 68 983 participants had relevant data for the analysis.

### Smoking status

We found an association between each additional smoking heaviness-increasing allele and increased odds of maternal smoking during pregnancy [odds ratio (OR) = 1.02, 95% confidence interval (CI) 1.01 to 1.03, *P = *0.002], and, as also reported by Yang and colleagues,^22^ lower odds of participants being an ever smoker (OR = 0.98, 95% CI 0.97 to 0.99, *P = *0.001), and lower birthweight of the participant [beta (β) = -0.01,95% CI -0.01 to -0.002]. These associations confirm the validity of the genetic instrument as a proxy for smoking status.

### Offspring schizophrenia

**Figure 2:**
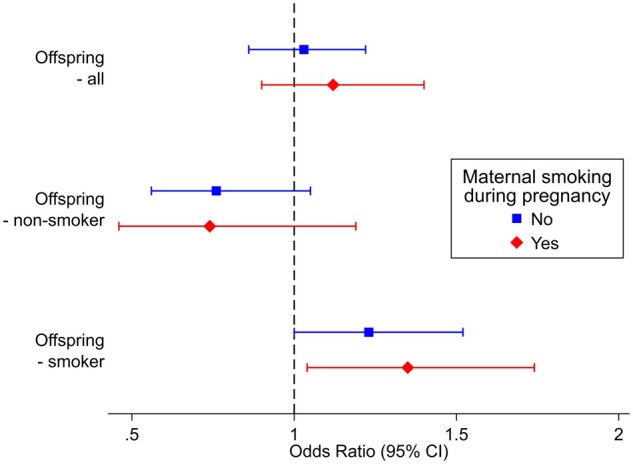
The association of participants’ rs16969968 genotype with diagnosis of schizophrenia stratified by maternal smoking status during pregnancy and their own smoking status

We found some evidence of an association between rs16969968 genotype and schizophrenia when stratifying on offspring smoking status (*P*_interaction_ = <0.001; Table 1). Among offspring who were never smokers, each additional risk allele acting as a proxy for maternal smoking heaviness appeared to have a protective effect (OR = 0.77, 95% CI 0.62 to 0.95, *P = *0.02), whereas among offspring who ever smoked, each additional smoking heaviness allele was associated with an increased odds of schizophrenia diagnosis (OR = 1.23, 95% CI 1.05 to 1.45, *P = *0.01). However, this effect was consistent regardless of maternal smoking status during pregnancy and there was no clear evidence of an interaction by maternal smoking status (*P*_interaction_ = 0.58–0.93; Table 2, Figure 2). This implies that any effects of smoking on schizophrenia risk is not due to maternal smoking in pregnancy.

**Table 1:** Association between rs16969968 genotype and offspring mental health when stratified by offspring smoking only, analyses not restricted on maternal smoking status

Outcome	Offspring smoking status	OR	95% CI	*P*-value	Interaction *P*-value	*N*
Schizophrenia	No	0.77	0.62 to 0.95	0.015	<0.001	161 372
	Yes	1.23	1.05 to 1.45	0.011		127 321
MDD	No	1.02	0.99 to 1.06	0.192	0.812	38 911
	Yes	1.02	0.98 to 1.06	0.377		30 072
Depression	No	1.01	0.98 to 1.04	0.727	0.553	161 372
	Yes	1.02	0.99 to 1.05	0.237		127 321

MDD, major depressive disorder; OR, odds ratio; CI, confidence interval.

**Table 2:** Proxy GxE analysis of maternal smoking heaviness during pregnancy on offspring mental health, adjusted for year of birth, offspring gender, top 10 principal components of ancestry

Outcome	Offspring smoking status	Maternal smoking around birth	OR	95% CI	*P*-value	Interaction *P*-value	*N*
Schizophrenia	All	No	1.02	0.87, 1.20	0.796	0.926	200 541
		Yes	1.03	0.83, 1.28	0.784		88 152
	No	No	0.80	0.62, 1.03	0.082	0.578	113 164
		Yes	0.70	0.47, 1.03	0.072		48 208
	Yes	No	1.21	0.99, 1.49	0.062	0.795	87 377
		Yes	1.27	0.97, 1.67	0.086		39 944
MDD	All	No	1.02	0.98, 1.05	0.346	0.694	48 272
		Yes	1.03	0.98, 1.07	0.263		20 711
	No	No	1.03	0.98, 1.07	0.218	0.654	27 534
		Yes	1.01	0.95, 1.08	0.747		11 377
	Yes	No	1.00	0.96, 1.05	0.967	0.218	20 738
		Yes	1.05	0.99, 1.13	0.128		9334
Depression	All	No	1.02	0.99, 1.04	0.253	0.446	200 541
		Yes	1.00	0.96, 1.03	0.930		88 152
	No	No	1.01	0.97, 1.05	0.626	0.253	113 164
		Yes	0.99	0.94, 1.05	0.790		48 208
	Yes	No	1.02	0.99, 1.06	0.225	0.638	87 377
		Yes	1.01	0.96, 1.06	0.769		39 944

MDD, major depressive disorder; OR, odds ratio; CI, confidence interval GxE, gene-by-environment.

Sensitivity analyses restricting to individuals who were homozygous at rs16969968 were consistent and found no strong evidence of an effect of maternal smoking heaviness during pregnancy on offspring schizophrenia (Table 3). The association between established risk factors and offspring schizophrenia was as expected. Increased years of education were associated with decreased odds of schizophrenia diagnosis (OR = 0.85, 95% CI 0.82 to 0.89, *P *<0.001), and greater genetic liability for schizophrenia was associated with increased odds of diagnosis (OR = 1.53, 95% CI 1.40 to 1.66, *P *<0.001) (Table 4). Although there was no strong evidence of an association between birthweight and schizophrenia diagnosis, the effect estimate was in the hypothesized direction (OR = 0.92 per kg birthweight, 95% CI 0.76 to 1.11, *P = *0.37). We also examined the association between the schizophrenia polygenic risk score (PRS) and years of education. Among controls we found some evidence of a small positive association (β = 0.01 per additional standard deviation in PRS, 95% CI 0.00 to 0.02, *P = *0.05). In the whole sample, evidence for a positive association was weaker and, when restricting to cases, the effect estimate suggested a negative association between the schizophrenia PRS and years of education (β = -0.11 per additional standard deviation in PRS, 95% CI -0.33 to 0.10, *P = *0.29) (Table 4). When restricting to cases, power was low and there was little evidence of an association.

**Table 3:** Sensitivity analysis restricting to rs16969968 homozygous individuals only. Proxy GxE analysis of maternal smoking heaviness during pregnancy on offspring mental health, adjusted for year of birth, offspring gender, top 10 principal components of ancestry

Outcome	Offspring smoking status	Maternal smoking around birth	OR	95% CI	*P*-value	Interaction *P*-value	*N*
Schizophrenia	All	No	1.03	0.86, 1.22	0.748	0.547	111 954
		Yes	1.12	0.90, 1.40	0.318		48 812
	No	No	0.76	0.56, 1.05	0.096	0.910	63 094
		Yes	0.74	0.46, 1.19	0.212		26 560
	Yes	No	1.23	1.00, 1.52	0.052	0.587	48 860
		Yes	1.35	1.04, 1.74	0.024		22 252
MDD	All	No	1.02	0.99, 1.06	0.175	0.555	27 098
		Yes	1.00	0.95, 1.06	0.852		11 504
	No	No	1.04	1.00, 1.09	0.076	0.247	15 435
		Yes	0.99	0.93, 1.06	0.840		6308
	Yes	No	1.00	0.95, 1.06	0.938	0.571	11 663
		Yes	1.03	0.95, 1.11	0.466		5 196
Depression	All	No	1.02	0.99, 1.05	0.271	0.327	111 954
		Yes	0.99	0.95, 1.03	0.690		48 812
	No	No	1.02	0.98, 1.06	0.332	0.211	63 094
		Yes	0.98	0.92, 1.04	0.430		26 560
	Yes	No	1.01	0.97, 1.06	0.522	0.937	48 860
		Yes	1.01	0.96, 1.07	0.719		22 252

GxE, gene-by-environment; MDD, major depressive disorder; OR, odds ratio; CI, confidence interval.

**Table 4:** Association between established risk factors for schizophrenia and offspring schizophrenia in UK Biobank

Exposure	Outcome	OR	95% CI	*P*-value	*N*
Education (years of schooling)	Schizophrenia diagnosis	0.85	0.82 to 0.89	<0.001	286 262
Genetic liability for schizophrenia	Schizophrenia diagnosis	1.53	1.40 to 1.66	<0.001	288 693
Birthweight (kg)	Schizophrenia diagnosis	0.92	0.76 to 1.11	0.374	173 402

		β	95% CI	*P*-value	*N*

Genetic liability for schizophrenia	Education (years of schooling): whole cohort	0.01	−0.001 to 0.016	0.072	286 262
Genetic liability for schizophrenia	Education (years of schooling): among controls	0.009	0.0002 to 0.017	0.046	285 740
Genetic liability for schizophrenia	Educational (years of schooling): among cases	−0.11	−0.33 to 0.10	0.290	522

OR, odds ratio; CI, confidence interval; β, beta.

### Offspring depression

**Figure 3:**
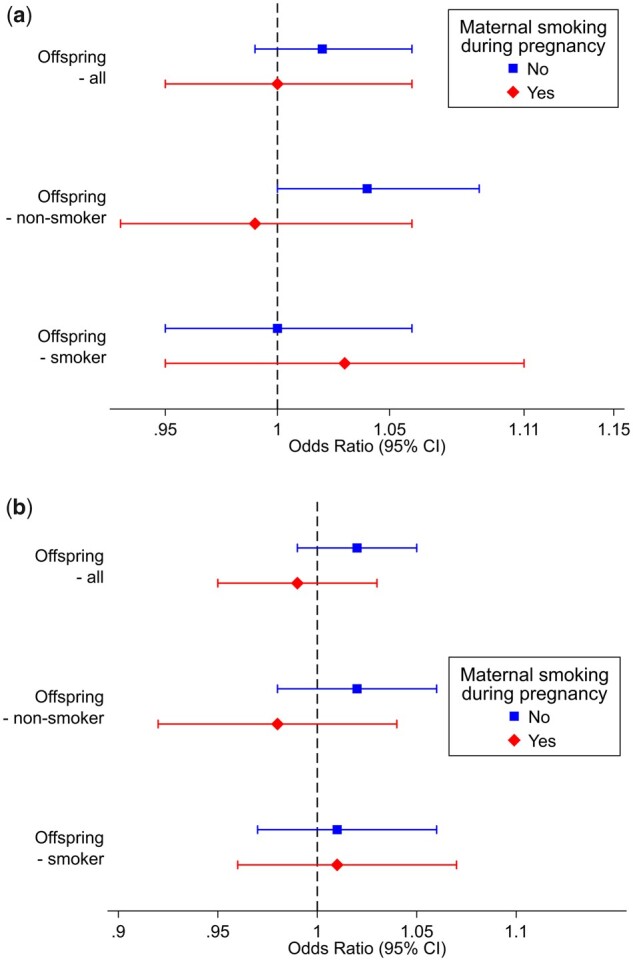
The association of participants’ rs16969968 with diagnosis of a) depression according to the mental health touchscreen questionnaire and b) depression according to the self-reported non-cancer illness code and ICD-10 codes. Both analyses were stratified by maternal smoking status during pregnancy and participants’ own smoking status. *ICD-10: International Classification of Diseases, Tenth Edition

We found no clear evidence of an association between rs16969968 genotype and either MDD or depression when stratifying on offspring smoking status, and there was no robust evidence of an interaction (Table 1). Effect estimates remained consistent when stratifying on maternal smoking status during pregnancy and we found no strong evidence of an association for either MDD or depression (Table 2, Figure 3). Sensitivity analyses restricting to homozygous individuals were consistent and identified no strong evidence of an association (Table 3).

## Discussion

In this study, we extended previous work by Wootton and colleagues that found a causal effect of own smoking on mental health, and applied methods developed by Yang and colleagues^22^ to investigate whether this was partially explained by causal effects of maternal smoking heaviness in pregnancy. Although we found evidence of an effect of offspring smoking heaviness on schizophrenia, these effects were consistent when stratifying by maternal smoking status, and we found no strong evidence of an effect of maternal smoking heaviness on offspring depression. Overall, we found little support for a causal effect of maternal smoking heaviness during pregnancy on offspring schizophrenia or depression or of own smoking on depression, but some evidence of a causal effect of own smoking status on schizophrenia diagnosis.

A recent review, combining 12 observational studies, suggests that both own smoking and prenatal tobacco smoke exposure may be independent risk factors for schizophrenia, with risk of schizophrenia increased by 29% after exposure to prenatal smoke.^9^ However, this review did not include studies such as sibling comparison designs, and as a result may be subject to familial confounding as described by Quinn and colleagues.^36^ Rather than representing a true causal effect, it is possible that the observed association between maternal smoking during pregnancy and offspring diagnosis of schizophrenia could be due to passive gene-environment correlation or other shared family factors.^36^ Recent sibling comparison studies that account for this familial confounding have found weaker effects of maternal smoking during pregnancy, which is in line with our findings.^14^^,^^19^

The association between rs16969968 genotype and schizophrenia when restricting to participants (offspring) who smoke is indicative of a causal role of smoking heaviness on schizophrenia. This is in line with recent findings reported by Wootton and colleagues,^8^ who used a two-sample Mendelian randomization approach and found strong evidence for a causal effect of both smoking initiation and lifetime smoking on schizophrenia. However, we also observe an inverse association between rs16969968 genotype and own smoking status. This is unexpected and could be due to selection bias, which could have implications for our interpretation of the association between rs16969968 genotype and schizophrenia. We know that there is evidence of selection based on smoking status, with the proportion of current smokers in UK Biobank lower than in the general population of the UK.^37^ Poor mental health is also linked with lower participation in cohort studies,^32^^,^^38^^,^^39^ with diagnoses of schizophrenia in the UK Biobank study (0.19%) being much lower than in the general population (∼1%).^31^ The increased participation of non-smokers and lower participation of individuals with schizophrenia could therefore induce a negative association between rs16969968 genotype and schizophrenia among non-smokers. Therefore, the association we observe between rs16969968 genotype and schizophrenia diagnosis may be a result of collider bias due to selection on study participation.

Depression is under less selection in UK Biobank and we did not observe an association between rs16969968 genotype and offspring depression when using either definition of depression. An alternative suggestion is that this negative association between rs16969968 genotype and schizophrenia among non-smokers is due to some underlying biology. Yang and colleagues^22^ found a similar effect on birthweight when stratifying on maternal and own smoking status and suggest potential pathways, including that mothers exposed as fetuses to intrauterine smoking may be less able to constrain the paternal impact. However, we did not have the data to investigate the effects on mental health across three generations.

Lifetime prevalence of schizophrenia in the general population is around 1%,^31^ but in the UK Biobank we observed a prevalence of 0.2%. In order to investigate the impact of collider bias on our results, we performed several sensitivity analyses. We first estimated the association between known risk factors for schizophrenia (educational attainment, schizophrenia polygenic scores, birthweight) and offspring diagnosis, all of which were associated in the direction we would expect and effect estimates were consistent with those identified in previous studies.^40^^,^^41^ When stratifying on diagnosis, the differences in association shown between genetic liability for schizophrenia and educational attainment between cases and controls fit with theories of stabilizing selection or cliff-edge fitness for schizophrenia persistence.^42^^,^^43^ Among those without a diagnosis there is a positive association between genetic liability for schizophrenia and educational attainment, whereas among those reaching the threshold for a diagnosis, the direction of effect appears to be in the opposite direction. We also looked at the interaction between maternal smoking heaviness and schizophrenia polygenic score and found no strong evidence of an association. Depression is under less selection in the UK Biobank cohort and should therefore be at less risk of collider bias, and analyses using offspring depression as the outcome also found no strong evidence of an association between maternal smoking during pregnancy and offspring mental health. This suggests that for both schizophrenia and depression, the observed association between maternal smoking during pregnancy and offspring outcomes may be due to residual confounding. This is consistent with findings by Taylor and colleagues who used a cross-cohort and negative control analysis to investigate the influence of maternal smoking during pregnancy on offspring depression.^21^

This work is subject to limitations. First, our analysis used offspring genotype at rs16969968 as a proxy for maternal genotype. Offspring genotype contains information inherited from fathers, reducing our power to detect interactions between the categories of maternal smoking status. As a sensitivity analysis, we excluded heterozygous individuals to ensure that the smoking heaviness-increasing allele was inherited from the mothers. The results remained consistent, with little evidence of an effect of maternal smoking heaviness.

Second, rs16969968 is a proxy for smoking heaviness across the lifetime, and its effects are not restricted to during pregnancy. It is possible that any association could act via other pathways, such as passive gene-environment correlation or other familial confounding factors.

Third, our analysis stratifies on both mother and offspring smoking status and as a result may be subject to collider bias.^37^ Previous simulations suggest this should have a minimal impact on our findings, although it is likely that the extent of collider bias is larger with respect to how schizophrenia and its underlying genetic liability influence entering the study than in these previous examples.^22^^,^^44^

Fourth, offspring schizophrenia is under-represented in UK Biobank, which could mean our results are subject to collider bias. However, we performed several sensitivity analyses to check the association of this phenotype with established risk factors and they behaved as expected. We also included depression as an outcome, which is under less selection in the sample, and our results were similar. The association between rs16969968 and schizophrenia in this study could be due to collider bias; however it is also consistent with findings by Wootton and colleagues, who found a strong association between lifetime smoking and schizophrenia diagnosis.

Fifth, our exposure and outcome measures may be misclassified. We used offspring smoking status as reported at recruitment; however, smoking status is not constant across the life course and it may have differed at critical developmental periods prior to illness onset. Maternal smoking during pregnancy was derived using offspring responses to a question that asked about smoking around the time of birth, rather than during pregnancy. There is evidence to suggest that offspring reports of maternal smoking during pregnancy have reasonable validity.^45^ Although the question relates to smoking around the time of birth, at the time of these pregnancies there was little guidance around the use of tobacco during pregnancy. The first report *Smoking and Health* was published by the Royal College of Physicians (RCP) in 1962,^46^ whereas 90% of our sample were born in (or prior to) 1963. Therefore, although asking about smoking during the time of birth may have resulted in increased misclassification of smoking status during pregnancy, the risk is likely to be minimal in this cohort. It is also well documented that maternal smoking during pregnancy is associated with lower offspring birthweight, including within UK Biobank.^47^ As shown by Yang and colleagues,^22^ the rs16969968 SNP is associated with lower birthweight among offspring of mothers reporting smoking during pregnancy. It therefore seems likely that misclassification was minimal and that the variant reflects maternal smoking heaviness during pregnancy. Schizophrenia diagnosis and one of the depression definitions were based on positive responses to two items, so it is possible that some individuals who did not respond to these items were incorrectly classified as controls. This misclassification could lead us to underestimate a true causal effect, should one exist.

Sixth, data on smoking initiation, schizophrenia and depression were collected retrospectively and we did not have ages at diagnosis and initiation for the majority of our sample. Where these data were available, 92% of individuals reported smoking prior to receiving a diagnosis of schizophrenia. Additionally, given that 90% of lifetime smoking is initiated between ages of 10 and 20,^48^ whereas the median age of onset for depression and schizophrenia occurs during early to mid-adulthood,^49^ it seems likely that for the majority of participants, smoking initiation would precede any diagnosis.

## Conclusion

Our findings are in line with those reported by Wootton and colleagues,^8^ which supported a causal effect of own cigarette smoking on schizophrenia diagnosis, although unlike Wootton and colleagues, we found little evidence of an association between own smoking and depression. We also found little evidence to suggest a causal effect of maternal heaviness of smoking during pregnancy on either offspring schizophrenia or depression.

## Ethics approval

UK Biobank is approved by the National Health Service National Research Ethics Service (ref. 11/NW/0382; UK Biobank application number 9142).

## Data Availability

The UK Biobank dataset used to conduct the research in this paper is available via application directly to the UK Biobank. Applications are assessed for meeting the required criteria for access, including legal and ethics standards. More information regarding data access can be found at [https://www.ukbiobank.ac.uk/enable-your-research].
